# Sleep Disorders in Children with Rett Syndrome

**DOI:** 10.3390/children12070869

**Published:** 2025-06-30

**Authors:** Christopher Harner, Thomas A. Gaffey, Shannon S. Sullivan, Manisha Witmans, Lourdes M. DelRosso, Mary Anne Tablizo

**Affiliations:** 1 College of Science, California State University, Turlock, CA 95382, USA; 2 Department of Psychology, University of Notre Dame, Notre Dame, IN 46556, USA; tgaffey@nd.edu; 3 Division of Pediatric Pulmonology and Sleep Medicine, Department of Pediatrics, School of Medicine Stanford, Stanford University, Lucile Packard Children’s Hospital, Stanford, CA 94305, USA; 4 Division of Pulmonology, Department of Pediatrics, University of Alberta, Edmonton, AB T6G 2R3, Canada; 5 Division of Sleep Medicine, Department of Internal Medicine, UCSF, Fresno, CA 93701, USA

**Keywords:** Rett syndrome, neurodevelopmental disorder, sleep disorder

## Abstract

Rett syndrome (RTT) is an X-linked neurodevelopmental disorder marked by neurological regression, autonomic dysfunction, seizures, and significant sleep and breathing abnormalities. About 80% of affected individuals, especially young children, experience sleep disturbances such as insomnia, sleep-disordered breathing, nocturnal vocalizations, bruxism, and seizures. Breathing irregularities during sleep—like apnea, alternating hyperventilation, and hypoventilation—are common, with both obstructive and central sleep apnea identified through polysomnography. This review focuses on the prevalent sleep disorders in children with Rett syndrome and highlights current recommendations for the management of sleep disorders.

## 1. Introduction

Rett syndrome (RTT) is a rare, progressive X-linked genetic neurodevelopmental disorder marked by behavioral and neurological regression, dysautonomia, seizures, and motor deficits. The disorder was first described by Dr. Andreas Rett in 1966 [1] and reported in the international community by Hagberg et al. in 1983 [2]. Classic Rett syndrome (RTT) is characterized by four primary neurological criteria: loss of purposeful hand movements, loss of acquired spoken language, an abnormal gait, and stereotypic or repetitive hand movements [3]. Classic RTT is additionally marked by a brief period of normal development, followed by loss of acquired skills such as hand use and speech, deceleration of head growth, and breathing irregularities [3]. Individuals with variant or atypical RTT meet at least 2 of these main neurological criteria, along with 5 out of 11 supportive criteria. Supportive criteria may include symptoms such as bruxism, breathing irregularities while awake, sleep disturbances, abnormal muscle tone, peripheral vasomotor instability, episodes of screaming or inappropriate laughter, scoliosis or kyphosis, cold and small hands and feet, reduced pain sensitivity, intense eye contact, and growth retardation [3]. Among atypical RTT, three subsets may be identified, including a preserved speech variant, a congenital variant, and an early seizure variant [3]. Although RTT predominantly affects females, there are rare cases in males with an additional X chromosome or somatic mosaicism [4].

RTT occurs in approximately 1 in 10,000 live female births [5] and accounts for up to 10% of cases of severe intellectual disability due to genetic causes in females [6].

Sleep disturbance is a critical aspect of the disorder, and understanding sleep in RTT contributes to a comprehensive understanding of this syndrome, given the negative physical and mental health impacts on affected individuals as well as their caregivers. Studies revealed that an estimated 80% of those with RTT experience sleep-related difficulties [7], underscoring the need for effective interventions. These sleep disturbances have negative effects on daytime behavior, cognition, and overall development of the child and contribute significantly to caregiver burden [8].

Despite sleep’s significant impact on child development and quality of life, evidence-based treatment options for sleep disorders in pediatric patients with RTT remain limited, with few achieving substantial improvements. This review synthesizes selected peer-reviewed research to examine prevalent sleep disturbances in children with Rett syndrome, objective findings in polysomnography (PSG), current management strategies, and provide evidence-based insights on its management for healthcare providers. Several studies suggested that there are variations in sleep problems associated with mutation type. Hence, we will start by describing the genetic mutations linked with RTT [7].

Articles were chosen based on clinical relevance, recency, and quality of methodology. This work is not intended as a systematic review, but it aims to comprehensively summarize key evidence and practical recommendations. Because of the paucity of evidence and research on RTT, case reports were also included in the treatment of sleep disturbances. This review further highlights the need for more registry-based comprehensive research to develop treatment options for this vulnerable population.

## 2. Genetics of Rett Syndrome

Pathogenic variants in the MECP2 gene located in the Xq28 chromosomal region were first associated with RTT in 1999. Over 900 mutations (pathogenic and benign) in MECP2 have now been identified, with substantial phenotypic variation [9,10]. The MECP2 gene is a key player in regulating DNA methylation and gene transcription, and thus mutations may have broad downstream consequences [11]. In particular, MECP2 plays important roles in neuronal development, dendritic branching, and brain morphology [12]. Functional impacts of mutations in MECP2 include alterations in synaptic function, neuronal connectivity, and gene expression patterns in brain regions implicated in RTT [13].

Most cases arise sporadically through de novo mutations. MECP2 mutations are associated with a spectrum of clinical presentations, ranging from asymptomatic female carriers with complete skewing of X chromosome inactivation [14] to males who develop severe neonatal-onset encephalopathy [3].

Research studies established that approximately 95% of classical RTT cases and 58–75% of atypical RTT cases have mutations in MECP2 [15]. Recent discoveries have unveiled mutations in loci other than MECP2, which have been linked to atypical RTT [3]. Mutations in genes such as cyclin-dependent kinase-like 5 (CDKL5) and forkhead box G1 (FOXG1) have been implicated in clinical presentations resembling RTT, underscoring the genetic heterogeneity of conditions that share similar clinical features [15,16]. Additionally, MECP2 mutations have also been identified without the clinical features of RTT, and it is important to note that MECP2 mutations are neither necessary nor sufficient for diagnosis, which remains clinical [3].

There is evidence that particular genetic mutations may be associated with heightened sleep dysfunction [17,18,19], though sleep complaints of one type or another are nearly universal in those with RTT. The associations between particular genetic mutations and specific sleep problems are addressed in the relevant sections of this paper. Understanding the underlying genetics and neurobiology of the disorder may help pave the way for the development of targeted therapies [20] to address specific mechanisms of dysfunction.

## 3. Sleep Disturbances in Rett Syndrome

Impaired sleep patterns are part of the supportive diagnostic criteria for RTT, and disturbed sleep has been noted from the earliest descriptions of the syndrome [21,22]. In some cases, sleep disturbances may be one of the earliest presenting symptoms. Approximately 80% of children with RTT experience sleep disturbances, with variable occurrence throughout life but generally a higher prevalence [7] in early childhood [17], consistent with the observation that heightened behavioral symptoms occur around the regression period [23]. Sleep disturbance is most prevalent in children up to 7 years of age, with a greater than 90% probability of any sleep disturbance. Thereafter, studies indicate some reduction in sleep disturbance in later childhood and adolescence [24,25]. Sleep disturbances in children with RTT include insomnia (both initiating and maintaining sleep), abnormal breathing in sleep, sleep vocalizations such as laughing and screaming, bruxism, sleep-related seizures, and excessive daytime sleepiness.

### 3.1. Disturbances in Sleep Initiation and Maintenance

Insomnia, defined as difficulty initiating or maintaining sleep, is a leading sleep disturbance for RTT patients [26,27,28]. In a study involving caregivers of 364 patients with RTT with confirmed MECP2 mutations, registered in the International Rett Syndrome Phenotype Database (InterRett), who completed the validated Sleep Disturbance Scale for Children (SDSC), findings indicated that over 80% of RTT patients experienced night wakings, and nearly half of this group experienced night wakings often. The median age of this sample was 14.5 years (range: 2.1–57.2 years), all but two were female, and 93.4% lived at home [19]. In this group, over 60% of the patients also experienced difficulty falling asleep. Younger children and those with the p.Arg294 mutation exhibited the most significant difficulties in sleep initiation and maintenance. Recent evidence showed that longer sleep onset latency is associated with impaired daytime interactivity among individuals with RTT which has underscored the important impacts of sleep disturbances on next-day function [29]. In a recent actigraphic study of 29 individuals with RTT and their caregivers, shorter total sleep times (TST), as well as low sleep efficiency, long sleep-onset latency, elevated wake after sleep onset (WASO), and fragmented sleep were demonstrated in both older and younger subjects [30]. A limitation of much of the current literature on sleep in RTT is that although sleep disruptions are commonly reported, associated or co-occurring findings are not well elucidated. At least one expert review reflects that, for example, disrupted sleep patterns may be related to hunger, gastroesophageal reflux, or constipation [31], but such associations have not been systematically measured in most cases.

Difficulty initiating sleep, night waking, and excessive daytime sleepiness in individuals with Rett syndrome (RTT) may be related to abnormalities in circadian rhythm regulation. Clinically, dysregulation of the sleep-wake cycle with delayed sleep onset and daytime sleeping is observed in RTT individuals. A study conducted by Piazza et al. [32] on 20 girls with RTT aged 1–32 years (mean age: 9 years; median age: 8 years) showed increased total sleep, significantly less nighttime sleep, delayed sleep onset, and significantly more daytime sleep. Nighttime sleep duration was negatively correlated with age, while daytime sleep duration was positively correlated with age. These findings suggest that there is almost an inverted pattern of sleep.

Excessive daytime sleepiness with daytime naps has been reported, particularly in older girls and women, likely from disrupted nighttime sleep and delayed sleep phase disorder. The sleep disturbances occurred across age and mutation groups in the Australian population-based sample. The prevalence of daytime napping increased with age [7]. Daytime napping was commonly reported in cases with p.R270X, p.R255X, and p.T158M mutations [7]. In the Boban et al. study described earlier, napping was reported in 42.8% of individuals, with over a third of individuals napping sometimes or often [19].

Evidence indicates that MECP2, CDKL5, and FOXG1 may play a role in regulating specific brain areas and neurotransmitters that affect the sleep-wake cycle [26]. Animal models of RTT have shown highly fragmented sleep and abnormal circadian rhythms with blunting of the circadian cycle [33]. The MECP2 gene is expressed in the suprachiasmatic nucleus, and photic stimulation causes phosphorylation [34]. There may be other genes involved that also affect the circadian rhythm, resulting in disorganization of the entire circadian rhythm both centrally at the suprachiasmatic nucleus as well as peripherally to the organs.

### 3.2. Abnormal Nocturnal Breathing

**Figure 1:**
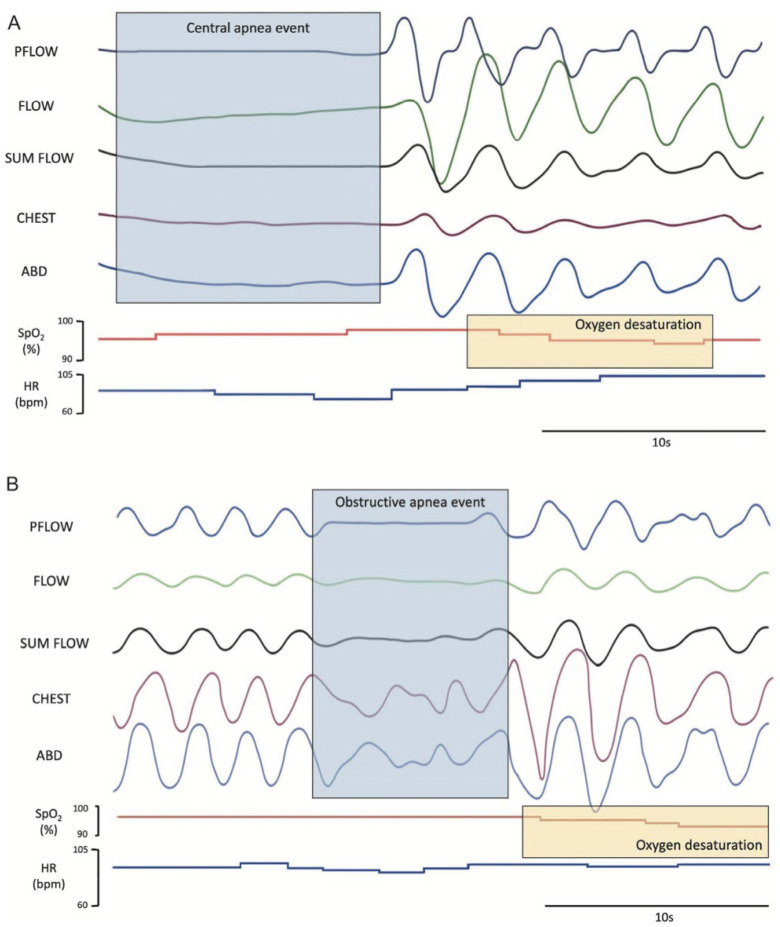
Sleep-disordered breathing in RTT [35]. Polysomnography studies on RTT have shown both obstructive (**B**) and central sleep apnea (**A**) with associated hypoxia [35].

The classical respiratory alterations of RTT include breath-holding and irregular breathing, with over 80% of affected individuals experiencing respiratory abnormalities during their lifespans [35]. MECP2 is expressed throughout the brainstem, including regions in the pons and medulla, which is important for control of breathing. It is thought that aberrant MECP2-associated transcriptional control leads to alterations in normal breathing patterns and classical breath-holding, hyperventilation, and overactive expiration in individuals with RTT [35,36]. Additionally, altered chemosensory responsiveness to hypoxia and hypercapnia has been identified in RTT, further complicating abnormal respiratory patterns. It is estimated that 75% of children with RTT develop breath-holding during wakefulness before 6 years of age, with hyperventilation following 2–3 years later [35]. It has been demonstrated that the uncoupling between breathing and heart rate control indicative of autonomic dysregulation is more pronounced in waking than in sleep in girls with RTT with alteration in the MECP2 gene. This dysregulation has been demonstrated in sleep as well and may represent a risk for sudden death in patients with RTT [37]. Abnormal breathing, including apnea, gasping, alternating hyperventilation, and hypoventilation, is common during sleep [38,39] in RTT, although there is variability between individuals and over time within individuals [40]. Polysomnography studies in RTT have shown both obstructive and central sleep apnea along with associated hypoxia (Figure 1A and B) [11].

In addition to respiratory control abnormalities, characteristics such as low tone may influence craniofacial development and scoliosis, which can complicate abnormal breathing in sleep. Abnormal breathing may lead to sleep fragmentation and awakenings, which exacerbate symptoms of insomnia and daytime symptoms, including mood and behavioral dysfunction. In one study, parents noted a greater impact of breathing abnormalities for individuals with the p.Arg294* variant [41], though the genetic variant did not seem to modify the Rett Syndrome Behaviour Questionnaire (RSBQ) score in another study [22].

### 3.3. Other Sleep Abnormalities (Vocalizations, Abnormal Movements, Bruxism, and Nocturnal Seizures)

Sleep-related vocalizations, movements, and bruxism are routinely observed in RTT. Among children with RTT in the 0–7-year age group, night laughing and night screaming have been reported to be extremely common, occurring at rates of 77% and 49%, respectively [24], with the prevalence and frequency of night laughter and screaming more likely in genotypes involving a large deletion of the MECP2 gene. After adjusting for age, the likelihood of experiencing any sleep disturbance was highest in individuals with large deletions (94%) and p.R270X (92%) and p.R294X (91%) mutations. Night laughing was especially prevalent in individuals with large deletions (90%) and the p.R168X mutation (78%), while persistent night laughing was moderately common among those with p.R106W (49%) and p.R168X (42%) mutations. Night screaming was most frequently reported in individuals with the p.R270X (57%), p.R306C (51%), and large deletion (51%) mutations [24].

Similarly, in the Boban et al. study described above, of 364 caregivers of individuals with RTT with a median age of 14.5 years, night laughing was reported for 58% of them, while night screaming, at 47.5% was slightly less commonly reported [19], and nocturnal bruxism (teeth grinding) was experienced by more than two thirds of individuals. Longitudinal evidence indicates some reduction in these symptoms over time, though persistence is common, especially among those with more severe and frequent symptoms at baseline [24]. 

Seizures are another common occurrence in RTT, with epilepsy occurring in 48–90% of affected individuals [42,43]. Nocturnal seizures may become more evident in later childhood or early adolescence [44], and seizure activity may be refractory and can become severe, including nocturnal electrical status epilepticus [45]. Boban et al. reported a rate of 52.8% of RTT individuals indicating the occurrence of nocturnal seizures, with 10.7% experiencing nocturnal seizures often. Patients with severe seizure activity were noted to be more likely to have poor sleep quality, suggesting that management of comorbidities is needed [19,26].

## 4. Sleep Impacts on Mental and Behavioral Health and Quality of Life

Addressing sleep disturbances is crucial, as they significantly impact mental and behavioral health in individuals with RTT. Insomnia and excessive daytime sleepiness showed the strongest associations with overall RSBQ scores and subscales related to mood, breathing, nighttime behaviors, and anxiety, reflecting the critical role of sleep in behavioral and mental health in RTT. Poor sleep negatively affects both mental health and quality of life [17]. Furthermore, both insomnia and daytime sleepiness have strong relationships with the RSBQ general mood subscale, as well as nighttime behaviors, breathing problems, and fear and anxiety subscales [17]. This link between sleep dysfunction and behavioral and emotional status in RTT is consistent with other studies, including evidence from the International Rett Syndrome Database demonstrating a relationship between poor sleep (insomnia and excessive daytime sleepiness) and heightened anxiety among 210 individuals with RTT aged 6–51 years [18]. Sleep disturbances in RTT also have a profound impact on overall quality of life. Longitudinal evidence indicates that quality of life in RTT has also been shown to be linked to sleep, with increased sleep disturbances associated with quality of life reduction [46]. Frequent sleep disturbances in individuals with RTT have been linked to later lower caregiver well-being scores [47], highlighting the long-term burden that disrupted sleep can place on families. In a recent study evaluating the frequency of sleep symptoms and burden on RTT individuals and their caregivers, sleep disturbances were again reported to be common (with 71.8% reporting sleep disturbances), but notably, the burden of illness was measured to be high for caregivers, being almost twice the rate for individuals with RTT, even though the overall symptoms were rated to be of mild-to-moderate severity. The authors concluded that the burden of sleep disturbances for caregivers was disproportionate to clinical severity [47]. Another explanation could be that clinical severity may be misrepresented based on the aspects that were evaluated.

## 5. Sleep Measurement and Objective Findings in Rett Syndrome

Common measures of sleep in children with RTT include sleep diaries (filled out by caregivers or family members), actigraphy, and in-lab polysomnography. Both sleep diaries and actigraphy offer the advantage of measuring sleep-wake cycles across many nights, commonly 2–3 weeks or longer. Although both sleep diaries and actigraphy [48] have been used to assess sleep in individuals with RTT, recently, the discordance between sleep diaries and actigraphy has been investigated, indicating 14.8% bedtime discordance and 22.6% wake time discordance of 45 min or greater between the two methods [49]. In the sample of 38 individuals (aged 2–26 years, mean of 13.1 years), missing data (instances where either diary entries or actigraphy data were incomplete or unavailable) were also common. Greater levels of missing data and discrepancies between diary-reported and actigraphy-recorded sleep patterns were correlated with higher clinical severity of Rett syndrome symptoms and a lower reported quality of life. The direction of difference between diaries and actigraphy was variable, and the study highlights limitations for both types of longitudinal sleep measures.

In contrast, polysomnography allows objective measurement of sleep features in a laboratory setting and is important for the detection of co-occurring parasomnia, sleep fragmentation, seizure activity and sleep-related breathing dysfunction and for identifying sleep or wake status and sleep architecture. Because studies are performed in a laboratory setting, some sleep features such as sleep onset latency and nighttime wakings may not reflect routine experience in the home setting. In addition, one night of monitoring may also not provide sufficient information for this complex sleep-wake dysregulation.

Polysomnography studies in RTT have shown both obstructive and central sleep apnea along with associated hypoxia (Figure 1). In a small polysomnography study of 13 pediatric patients (mean age: 10 years) with RTT, all individuals exhibited episodes of hyperpnea followed by pauses while awake, and 9 of the subjects had obstructive sleep apnea (OSA) with a mean AHI of 8.77 ± 8.82. Among these, 4 of these patients had severe OSA, and 1 had severe OSA combined with central apnea. The most common presenting symptoms were snoring (77%) and witnessed apnea (53.8%) [50]. In a larger study aggregating PSG data from 11 studies meeting eligibility criteria and reporting on 69 individuals with RTT and a mean age of 8.9 +/− 5.2 years, the mean AHI was found to be 12.25 ± 23.89, roughly split between central and obstructive apneas, with an oxygen desaturation index of 18.62 ± 31.9 [51]. Epilepsy and scoliosis appeared to be common in this study group, though data on these co-occurring conditions were missing for nearly half the aggregated sample.

Polysomnographic recordings in RTT also indicate reduced sleep efficiency and differences in sleep architecture, in addition to the breathing abnormalities discussed above. In the aggregated sample of 69 individuals with RTT and published PSG data, sleep efficiency (SE), which represents the percentage of time asleep from lights off to lights on in a laboratory setting, was reduced compared with same-age typically developing individuals, and impaired sleep continuity was also reported [51]. PSG evidence of disrupted sleep in children with RTT has also been reported by Carotenuto et al., who used a case-control approach for 13 individuals with RTT (mean age: 8.1; SD: +/− 1.4 years) and found significantly increased stage shifts per hour, awakenings per hour (averaging 6.3 versus 1.9 awakenings per hour in unaffected individuals), and elevated Wake After Sleep Onset (WASO) rates [52]. Similarly, SE was reported to be reduced in a study of 17 girls (mean age: 9.5 ± 2.8 years) [40] at 66 ± 19%, with only 3 girls presenting SE values above 80%, and WASO was also elevated.

Sleep architecture in RTT has also been reported to be altered, characterized by a decrease in rapid eye movement (REM) and a commensurate increase in non-REM (NREM) sleep, particularly N3 (slow wave), though concomitant sleep breathing disorders were seen in this study group, with a mean apnea-hypopnea index of 19 ± 37 events per hour [38]. Zhang and Spruyt, in their aggregated report on 74 PSGs of RTT individuals across 11 studies, also found increased N3 sleep and reductions in REM sleep, hypothesizing that aberrant sleep cycling, possibly associated with a poor REM “on switch” and preponderance in slow and high-voltage sleep, characterizes sleep in RTT. However, in this study, concomitant sleep-disordered breathing was also quite common, and the authors reported a mean AHI of 11.92 ± 23.67/h in these aggregated cases [51]. The increase in the percentage of slow-wave sleep in these reports contrasts with an earlier report on reduced slow-wave sleep in RTT (though increased delta power was observed within N3 sleep cycles) involving a retrospective case-control study of 10 girls with RTT (age range: 2–9 years) [53]. SE was again reduced in RTT compared with the unaffected girls.

Elevated periodic limb movements (PLMs) have also been noted in PSG, with Carentuto reporting an elevated mean periodic limb movement index (PLMI) in 13 girls with RTT compared with matched controls, with the girls with RTT having a PLMI of 9.5 events per hour, compared with 2.8 events per hour for the controls [38,52].

Recent work by Davis et al. (2023) [54] identified altered phase-amplitude coupling (PAC) during slow-wave sleep (SWS) as a potential circuit-level biomarker of sleep-wake dysregulation. In a cohort of children with RTT, PAC between slow-wave and theta (SW:T) and slow-wave and spindle (SW:S) activity showed disrupted topography, with ectopic coupling outside the typical vertex region. This abnormal coupling pattern was not only distinct from typically developing controls but also correlated positively with overall clinical severity, as measured by the Clinical Global Impression-Severity scale, which assesses communication, motor function, seizures, and autonomic features. These findings suggest that altered SWS dynamics may reflect broader neurodevelopmental impairment in RTT and could serve as a noninvasive biomarker for disease severity and progression [54].

Heart rate variability in Rett syndrome, as observed via PSG, is characterized by reduced overall variability and a shift toward sympathetic dominance and vagal withdrawal. This was demonstrated in a 2024 study where individuals with RTT exhibited a consistent shift in sympatho-vagal balance toward sympathetic dominance and vagal withdrawal during both wakefulness and all sleep stages. This alteration in autonomic regulation in RTT has potential implications for cardiovascular health. These findings underscore the need for further research to explore the broader physiological and clinical consequences of this dysautonomia [55].

Taken together, objective findings based on PSG in individuals with RTT demonstrated significant sleep abnormalities and poor sleep quality. Common findings include reduced SE, increased arousals and WASO, elevated PLMs, increased N3 and disrupted sleep architecture, with diminished REM sleep. Abnormal breathing patterns—characterized by episodes of hyperpnea and apnea and both obstructive and central apneas with associated hypoxemia—are also frequently observed. These findings underscore the pervasive and multifaceted nature of sleep-disordered breathing in RTT.

## 6. Sleep-Directed Therapies

Overall management approaches in RTT involve addressing symptoms and, when possible, preventing progression. Therapies for RTT involve multiple systems and targets and are reviewed in detail elsewhere [31], though it is worth noting that some therapies targeting other disease manifestations may themselves have influences on sleep (for example, antiepileptic or mood-targeted medications). In March 2023, trofinetide, a synthetic analog of a naturally occurring brain peptide and insulin-like growth factor 1 (IGF-1)-related compound thought to act on NMDA receptors, was approved by the United States Food and Drug Administration (FDA) [56] for treating children aged 2 years or older, and there are several other agents currently in clinical trials [57,58]. Trials of trofinetide have demonstrated good efficacy in improving scores on the Clinical Global Impression-Improvement scale and RSBQ, with a specific effect only on the General Mood subscale and Repetitive Face Movement subscale, but no effects were documented on other subscales [59]. While sleep-specific outcomes were not primary endpoints in trofinetide’s clinical trials, improvements in Rett Syndrome Behavior Questionnaire (RSBQ) subscales—particularly general mood and nighttime behaviors—suggest potential indirect benefits for sleep. A 2023 review by Bricker and Vaughn also described a case report in which a patient with RTT experienced sleep improvement after initiating trofinetide, though the authors noted that further studies are needed to evaluate trofinetide’s direct effects on sleep in this population [60].

Managing sleep complaints and disorders in RTT requires both a pragmatic and multi-modal approach, including behavioral therapies, appropriate medications, and addressing co-morbid conditions that impact sleep. These strategies should be age-based and focused on enhancing sleep consistency and stability, as well as quality and daytime status. Despite growing interest in effective interventions for sleep disorders in RTT, an outcome-driven evidence base for specific sleep-targeted therapies in RTT is sparse, hindering definitive treatment algorithms. At least one observational study using data from 320 families in the Australian Rett Syndrome Database examined the long-term management of sleep disturbances in Rett syndrome over a 12-year period. Despite limitations—such as missing data on specific sleep disorders (e.g., napping, seizures, apnea, and bruxism), potential overestimation of prevalence, and limited assessment of treatment effectiveness the study highlights the persistent and burdensome nature of sleep disturbances in RTT. It also reveals that clinical management remains inconsistent and largely based on clinician experience, emphasizing the need for individualized care strategies [24].

More recently, expert-led consensus guidelines for primary providers on managing RTT was published in 2020 [61], and comprehensive care guidelines, which include recommendations for sleep, can also be found on the International Rett Syndrome Foundation (IRSF) website [62]. The 2020 guidelines indicate that at a baseline visit and every six months, care providers for patients with RTT should review sleep onset and maintenance, respiratory symptoms, and the frequency of nocturnal interventions by caregivers, as well as review bed and bedroom safety [61]. Similarly, IRSF guidelines recommend screening for disrupted sleep by asking specific questions about bedtime, the time required to fall asleep, wake time, nocturnal awakenings, sleep disturbances, and the sleep environment [62] The IRSF guidelines also recommend screening for snoring, respiratory pauses, gasping, restless sleep, and abnormal behaviors and recommend referring to a specialist as appropriate [62]. Additional details on treatments for specific sleep conditions are summarized below.

### 6.1. Disturbances in Sleep Initiation and Maintenance

Behavioral strategies remain the cornerstone of managing sleep disturbances and insomnia and are considered first-line treatments. Given the high prevalence of sleep disturbances in individuals with RTT, foundational interventions such as establishing consistent bedtime routines with regular bedtimes and wake times and optimizing the sleep environment—minimizing noise, reducing light exposure, and maintaining a cooler and comfortable room temperature—are essential. While these interventions have not been rigorously studied specifically in the RTT population, they are widely used and serve as a key starting point for addressing sleep disturbances, especially in pediatric neurodevelopmental disorders [63,64]. In addition, consistent exposure to morning light, reducing maladaptive sleep associations, reinforcing limit-setting behaviors, and promoting structured daytime routines that incorporate physical activity and regular meals help promote normal sleep [26,44].

If daytime naps are employed, which may be age-appropriate or a useful tool for excessive daytime sleepiness, then they should be scheduled as part of a routine and undertaken while considering nocturnal sleep patterns [32]. Detailed sleep hygiene and behavioral sleep strategies are available [65,66], and it is important to note that in children with RTT, some trial and error of what works best and individualized care for each child may be necessary to find optimal strategies. In a report by Boban et al. which analyzed caregiver responses for 364 RTT individuals, including 274 taking no medications for sleep, sleep behavioral and hygiene strategies were employed by approximately two thirds of their families, and better use of sleep hygiene practices was associated with significantly lower odds of moderate or major impacts on the family (odds ratio: 0.60; 95% confidence interval: 0.37–0.98) and lower scores for disordered initiation and maintenance of sleep [28]. Similarly, in an older small study on three individuals with RTT, the behavioral strategy of bedtime fading was effective in advancing bedtime and promoting more regular sleep patterns by increasing the appropriate nighttime sleep duration, reducing inappropriate daytime sleep, and reducing problematic nighttime behaviors (e.g., nocturnal awakenings) [32]. This experience is reinforced by retrospective analysis indicating that among children with rare genetic neurodevelopmental disorders, implementing behavioral strategies improved sleep disturbances in the substantial majority of the patients studied, and improvements were maintained at long-term follow-up [67,68]. In RTT in particular, qualitative studies involving caregivers have indicated that a stable sleep routine improves sleep disturbance in individuals with RTT and establishes daily routines with reduced daytime stress [67].

While sleep hygiene is foundational, and behavioral therapy is accepted as first-line therapy for sleep disturbances, not all individuals with RTT will achieve adequate sleep without additional intervention, and pharmacological intervention may be considered. Given that individuals with RTT may also have respiratory abnormalities, abnormal cardiorespiratory autonomic function, and prolonged QTc intervals, caution is advised regarding safety when selecting pharmacologic agents for sleep disturbances [63].

The use of melatonin in RTT for sleep dysfunction was first reported several decades ago in a study on 4 weeks of oral melatonin administration at a dose of 2.5–7.5 mg (depending on body weight) among nine subjects with RTT in a placebo-controlled randomized crossover trial [69]. In this study, sleep onset latency (SOL) was reduced by 19.1 +/− 5.3 min, and the total sleep time (TST) and SE improved in those with worse baseline values. It should also be noted that the majority of the girls in this study were concomitantly taking anti-epileptic medications. More recently, the use of melatonin was reported to be associated with significantly improved sleep quality in RTT children in a survey study including 287 parent respondents [27]. It is worth noting that in the United States, melatonin supplements are available over the counter, and recent studies indicate large discrepancies between the labeled and actual melatonin content of many commercially available products. For example, one study found a range of actual content of melatonin that was 74–347% of the labeled content for 25 melatonin gummy products [70].

A variety of prescription medications (see Table 1) have also been reported for sleep disturbances in RTT, though little evidence exists to support specific pharmacologic therapies, and no evidence-based guidelines exist. Despite the lack of evidence, prescribing medications to help manage sleep symptoms on an individual basis is relatively common [71]. Medications used off-label and reported in RTT patients to manage sleep symptoms include (not in order of preference) clonidine, an α2-adrenoreceptor agonist; GABA-agonists such as clonazepam or zolpidem; and gabapentin [4]. Notably, clonidine and some other sedating agents may pose cardiac risks in individuals with Rett syndrome, particularly those with MECP2 mutations. These risks include QT interval prolongation, which can increase the likelihood of life-threatening arrhythmias and has been implicated in cases of sudden unexpected death in RTT. Trazodone, an atypical antidepressant that inhibits the uptake of serotonin and also has antihistamine activity and α1-adrenergic antagonism, may have the risk of QTc-interval prolongation [63], a consideration in RTT. Consensus guidelines for the care of RTT have suggested the consideration of melatonin to assist with the initiation of sleep and trazodone or clonidine to help maintain sleep [61]. Boban et al.’s survey conducted among 364 caregivers of children with RTT found that overall, 90 were on sleep medications, with the most common being 42 individuals on melatonin (either alone or as polytherapy), while 14 (3.9%) were on clonidine monotherapy, 12 (3.3%) were on trazodone monotherapy, with 15 (4.1%) reporting other medications for sleep (clonazepam, as well as other benzodiazepines and GABA-agonist hypnotics, chloral hydrate, diazepam, oxazepam, antihistamines such as diphenhydramine, cyproheptadine, hydroxyzine, antiepileptics, antipsychotics, antidepressants, and others including dextromethorphan, baclofen, magnesium, and medicinal cannabis), and 21 (5.8%) were on polytherapy [28]. The specifics on approaches for considering the more commonly encountered of these therapies are available [63,72], and consideration of off-target impacts and side effects in light of individual co-existing conditions is key. Other sedating medications such as atypical antipsychotics, antidepressants, antihistamines, and chloral hydrate are used less frequently but have been reported in children with neurodevelopmental disorders, particularly in cases of refractory insomnia [63]. An older study of the supplement L-carnitine reported an impact on sleep in RTT in an open-label trial with 21 girls and women with RTT (age range: 7–41 years; mean age of 14.4 years and median age of 10 years) compared with a control group of 62 individuals with Rett of a similar age for a 6 month period. Compared with the controls, treatment with L-carnitine led to significant improvements in sleep efficiency (*p* = 0.027), especially in the subjects with a baseline sleep efficiency of less than 90% [73]. In this investigation, daytime energy levels also improved. However, a follow-up investigation involving L-carnitine for sleep is lacking.

Pharmacology and considerations for the use of these agents have been published, though overall, there is little evidence for outcomes, and the likelihood of adverse events [63,71] must be evaluated [4]. Specialist involvement is recommended in approaching prescription medication management.

Managing co-existing conditions is an important aspect of improving sleep quality and insomnia symptoms. Evaluation and treatment of co-existing GERD and gastrointestinal dysmotility and constipation, which are incredibly common in RTT [74], as well as anxiety and mood disturbance, nocturnal breathing abnormalities, and restless legs syndrome, may contribute significantly to improved sleep quality. Alongside this awareness is the possibility that certain medications used to manage comorbid conditions may further impact sleep, sometimes negatively. Effective management of these medical issues, coupled with physician awareness of the potential sleep-related side effects of prescribed therapies, is essential for improving overall sleep outcomes.

Managing mood disturbances can significantly improve sleep. One case report [75] suggests the utility of selective serotonin reuptake inhibitors (SSRIs) in combination with a serotonin 1A agonist to manage mood issues and self-aggressiveness. One case report involved an 11-year-old girl with RTT who presented with a year-long history of sleep disturbances and self-harming behaviors. She had severe sleep disturbances, often sleeping only 4 h/night, which did not improve while on anti-migraine, anti-epileptic medications, risperidone, olanzapine, or clonidine. A trial of escitalopram yielded immediate improvements, with reduced self-harm and improved sleep (11 h nightly) lasting 3 months. However, SSRI-induced bruxism emerged, necessitating a dose reduction, which worsened behavioral issues and sleep disturbances [75].

### 6.2. Sleep-Related Movement Disorders

Sleep-related movement disorders, particularly restless legs syndrome (RLS), warrant consideration in the sleep evaluation of individuals with RTT, especially given the high prevalence of iron deficiency in this population. Expert consensus guidelines and those from the International Rett Syndrome Foundation recommend laboratory assessment for iron status, including fasting ferritin, serum iron, total iron-binding capacity, and transferrin saturation, when symptoms suggestive of RLS are present [61,62]. RLS is characterized by an urge to move the legs at rest, often accompanied by unpleasant sensations, with symptoms worsening in the evening and improving with movement, yet in young children, non-verbal children, or children with RTT, diagnosing RLS presents unique challenges, in which case supportive factors such as behaviors observed by the parent or caregiver, family history of RLS, personal history of anemia, and periodic limb movements of sleep (PLMS) on polysomnography may provide support for the diagnosis of pediatric RLS [76]. Importantly, iron deficiency is a known risk factor for RLS, and studies on RTT have reported iron deficiency anemia or depleted ferritin levels in up to 20% of individuals with RETT, further supporting routine screening [77]. The current guidelines for the treatment of RLS in children recommend iron supplementation as a first-line treatment [78].

### 6.3. Sleep-Related Breathing Disorders

Fu et al. recommended conducting an overnight sleep study for RTT individuals with snoring or pauses in breathing [61]. More recently, Italian expert consensus guidance on managing respiratory complications of RTT likewise states that nocturnal polygraphy and transcutaneous carbon dioxide monitoring are indicated in patients with congenital RTT and all RTT individuals presenting one or more of the following symptoms: snoring, obstructive apnea, hypotonia, or scoliosis [79]. If disordered breathing is identified, Cherchi et al. recommend noninvasive ventilation as tolerated, with consideration of noninvasive ventilation for all RTT patients with sleep respiratory disorders and those with hypotonia with associated hypercapnia. Beyond noninvasive ventilation, other therapeutic options have been contemplated [79]. For example, in light of the high degree of variability in breathing abnormalities even in the same individuals over time, it has been suggested that adenotonsillectomy be considered for sleep-related breathing disorders even when obstructive apneas do not predominate if they are present, and this therapy appeared to be reasonably common among children with RTT referred to a sleep specialist in a single-center retrospective chart review study [39,50]. That said, adenotonsillectomy is not fully curative, and persistent abnormal breathing has been reported [38].

Other reported therapies include medications such as acetazolamide and nasal steroids [50]. A retrospective study examined the use of acetazolamide, a carbonic anhydrase inhibitor, for treating SRBD in two patients. Both started at 5 mg/kg/dose, with serum bicarbonate levels monitored weekly, targeting ~15 mEq/L. The first patient, with an AHI of 21.2 events/h (mostly obstructive but suspected to have a central component), showed complete resolution of respiratory events (AHI: 0.3) after 17 months and no longer required daytime naps. The second patient, treated for breath-holding spells and nighttime apnea, initially required a dose increase to 8 mg/kg/dose (250 mg) to stabilize bicarbonate levels at 19 mEq/L. The breathing abnormalities resolved, and acetazolamide was stopped after 3 years of clinical improvement [50]. In the same retrospective study, a patient with mild OSA (OAHI: 2.8) was treated with nasal mometasone spray for one year, leading to improved snoring and reduced daytime naps. Despite no change in AHI on a repeat PSG three years later, the patient remained symptom-free [50].

SSRIs have also shown potential in improving respiratory abnormalities during sleep and wakefulness in individuals with RTT. Rodent models suggest that SSRIs can induce MECP2 gene expression and restore CO_2_ chemosensitivity, which is impaired in RTT patients [80]. In Mecp2-null male mice, citalopram (an SSRI) restored CO_2_ sensitivity, highlighting a therapeutic approach for RTT-related respiratory issues. While research studies on the use of SSRIs in RTT are lacking, case reports indicate promising results. In one case, an 11-year-old girl with hyperventilation and apneic attacks was successfully treated with 10 mg fluoxetine (an SSRI) twice daily, which significantly reduced hyperventilation and breath-holding episodes, and further improvement occurred after adding buspirone [81]. Another case reported on an 11-year-old female RTT patient with severe respiratory symptoms, including frequent apneic events during sleep, who was treated with tandospirone (a 5-HT1A agonist) and fluvoxamine (an SSRI) and experienced reduced apneic events, improved hand stereotypy, and reduced dysphagia [82]. These findings suggest that serotonergic agents may help address respiratory issues and associated behaviors in RTT linked to impaired serotonergic brain transmission. However, formal research studies are needed to confirm their efficacy.

This finding highlights a potential therapeutic approach for addressing respiratory abnormalities in RTT. As breathing disorders are common in RTT, increased interest in these agents has arisen [4].

Finally, research into neuromodulation suggests that substances that enhance GABAergic mechanisms may reduce breath-holding events and breathing irregularities in RTT and may restore chemosensitivity [35,80,83], suggesting promising avenues for the future.

### 6.4. Additional Recommendations

Given that individuals with Rett syndrome (RTT) may live into the fifth decade of life [84], often with a wide range of complex medical comorbidities—including autonomic dysregulation, motor impairments, epilepsy, swallowing difficulties, sleep disturbances, respiratory abnormalities, and gastrointestinal and orthopedic complications—there is a critical need to improve care strategies aimed at enhancing the quality of life for affected individuals.

Non-pharmacological strategies to manage problematic sleep in children with developmental disabilities, including RTT, are increasingly recognized as essential components of care. A comprehensive review by Spruyt and Curfs [85] evaluated 90 studies involving over 1400 children, with nearly half of the studies focusing on syndromes such as RTT. The most common interventions included bedtime routines, sleep scheduling, behavioral reinforcement techniques, and environmental adjustments such as sleep hygiene and sleep ecology. The review also highlighted the benefit of modifying the sleep environment (e.g., weighted materials, sensory-friendly bedding, and dim lighting) to promote continuous and effective sleep. Sleep ecology—including use of textured blankets, swaddling, and protective railings—was addressed in 21 studies, with success reported in two-thirds of them. Importantly, individualized interventions in the home setting were the most commonly reported and successful ones [85].

A recent preclinical study by Hung et al. demonstrated that music-based interventions improved social behavior and modulated neurobiological pathways in *mecp2* null/y mice, a model of RTT [86]. It also improved breathing patterns, and decreased the frequency of epileptic seizures. Specifically, mice exposed to daily music sessions showed enhanced social novelty behaviors and increased expression of brain-derived neurotrophic factor (BDNF) mRNA in the prefrontal cortex, along with elevated BDNF protein levels in the hippocampus. The intervention also upregulated FNDC5 gene expression, a known upstream modulator of BDNF, independent of TrkB signaling. These findings suggest that music may ameliorate RTT-associated social deficits through neuroplastic mechanisms involving BDNF/FNDC5 pathways. Although further studies in humans are needed, this study supports incorporating music-based therapies as a complementary approach to enhance social functioning, improve breathing pattern and emotional regulation in individuals with RTT and potentially alleviate sleep disturbances in individuals with Rett Syndrome.

Although a definitive cure for Rett syndrome (RTT) remains elusive, recent advancements in gene therapy have opened promising avenues for treatment. Approaches aimed at restoring normal *MECP2* gene function—the primary gene implicated in RTT—are showing particular promise, including the potential to improve associated sleep disturbances. In addition, ongoing research into pharmacological therapies targeting downstream pathways affected by *MECP2* mutations offers hope for symptom relief and meaningful improvements in quality of life for individuals with RTT [16].

**Table 1:** Medications used on RTT patients based on small studies, parent surveys, and case reports.

Drug Name	Class or Mechanism	Common Indication	Evidence in RTT Studies	Considerations in RTT
Melatonin	Synthetic exogenous hormone; regulates circadian rhythm	Insomnia, delayed sleep phase onset	Most commonly used in RTT based on parent survey [27,28]. One randomized crossover trial showed decreased SE and increased TST [69].	OTC, variable dosing practices
Clonidine	α2-adrenergic agonist	FDA approved for ADHD, >6 years old; off label for insomnia, RLS: indicated for antihypertensive in adults	Often used off-label as sedative in neurodevelopmental disorders [4,28].	Can lower blood pressure
Trazodone	Serotonin antagonist and reuptake inhibitor	Antidepressant for adults, off-label for insomnia	Mild sedative effects; discussed in behavioral comorbidity context [28,61].	QTc prolongation risk; priapism, limited pediatric data [63,68]; no FDA indication for pediatric patients
Gabapentin	GABA analog	Anticonvulsant, RLS, neuropathic pain, off -label for insomnia	Occasionally used as sedative for comorbid conditions in RTT with RLS and seizures; few data specific to pediatric RTT [4].	Sedation and mood effects should be monitored [4]
Clonazepam, diazepam, oxazepam	Benzodiazepine; GABA-A receptor agonist	Anticonvulsant, insomnia, anxiety, muscle relaxant	Based on parent survey, for dual use as sedative and for epilepsy management [4,28].	Dependence risk; respiratory depression in high doses [4]
Diphenhydramine	First-generation antihistamine	Allergies, off label OTC sleep aid	Based on parent survey, used as sedating option in refractory insomnia in 1 study [28].	Can cause paradoxical agitation; not for chronic use [28]
Hydroxyzine	First-generation antihistamine	Sedation premedication for procedure, anxiety, pruritus (approved for pediatric use)	Based on parent survey, used for insomnia [28].	Hypersedation, stupor, nausea, and vomiting
Cyproheptadine	Antihistamine	Allergies, appetite stimulant	Based on parent survey, used for insomnia [28].	Daytime sleepiness, weight gain
Escitalopram	SSRI	Antidepressant for >12 years old, general anxiety disorder for adults	One case report shows dramatic sleep and mood improvement in RTT [75].	Bruxism reported; dosing must be carefully titrated
Carnitine	Amino acid and nutritional supplement	Nutritional supplement	Open-label trial with 21 subjects showing improved SE and TST [73].	Older study and no follow up, 2001
Trofinetide	Synthetic analog of a naturally occurring brain peptide; insulin-like growth factor 1 [63,64]	Indicated for RTT for >2 years old; proposed mechanism of action is to promote synaptic maturation	Improved scores on RSBQ and clinical Global Impression Improvement scale. One case report with sleep improvement [59].	No direct evidence of improved sleep

These medications were used off-label and with no evidence to support their pharmacologic use in RTT. Their use should be carefully tailored to individual patient needs and comorbidities and monitored by clinicians familiar with RTT.

## 7. Conclusions

Children with RTT face a significantly increased risk of sleep disturbances, which impact not only the affected children but also their caregivers. These sleep issues underscore the need for further research to develop patient-specific treatments, particularly in the era of precision medicine. Sleep-related disturbances in RTT are diverse, necessitating interventions that address multiple aspects of sleep dysfunction [32]. By unraveling these complexities, researchers, clinicians, and families can work collaboratively to advance our understanding of the disorder and pave the way for more effective diagnostic, therapeutic, and supportive interventions. As we continue to learn more about RTT, we inch closer to improving the lives of those affected by this condition by improving their sleep.

## References

[1] Rett A. (1966). On a unusual brain atrophy syndrome in hyperammonemia in childhood. Wien. Med. Wochenschr..

[2] Dunn H.G. (2001). Importance of Rett syndrome in child neurology. Brain Dev..

[3] Neul J.L., Kaufmann W.E., Glaze D.G., Christodoulou J., Clarke A.J., Bahi-Buisson N., Leonard H., Bailey M.E.S., Schanen N.C., Zappella M. (2010). Rett syndrome: Revised diagnostic criteria and nomenclature. Ann. Neurol..

[4] Persico A.M., Ricciardello A., Cucinotta F. (2019). The psychopharmacology of autism spectrum disorder and Rett syndrome. Handb. Clin. Neurol..

[5] Laurvick C.L., de Klerk N., Bower C., Christodoulou J., Ravine D., Ellaway C., Williamson S., Leonard H. (2006). Rett syndrome in Australia: A review of the epidemiology. J. Pediatr..

[6] Armstrong D.D. (1997). Review of Rett syndrome. J. Neuropathol. Exp. Neurol..

[7] Young D., Nagarajan L., de Klerk N., Jacoby P., Ellaway C., Leonard H. (2007). Sleep problems in Rett syndrome. Brain Dev..

[8] Corchón S., Carrillo-López I., Cauli O. (2018). Quality of life related to clinical features in patients with Rett syndrome and their parents: A systematic review. Metab. Brain Dis..

[9] Amir R.E., Van den Veyver I.B., Wan M., Tran C.Q., Francke U., Zoghbi H.Y. (1999). Rett syndrome is caused by mutations in X-linked MECP2, encoding methyl-CpG-binding protein 2. Nat. Genet..

[10] Townend G.S., Ehrhart F., van Kranen H.J., Wilkinson M., Jacobsen A., Roos M., Willighagen E.L., van Enckevort D., Evelo C.T., Curfs L.M.G. (2018). MECP2 variation in Rett syndrome-An overview of current coverage of genetic and phenotype data within existing databases. Hum. Mutat..

[11] Zhang X., Smits M., Curfs L., Spruyt K. (2022). Sleep Respiratory Disturbances in Girls with Rett Syndrome. Int. J. Environ. Res. Public Heal..

[12] Lombardi L.M., Baker S.A., Zoghbi H.Y. (2015). MECP2 disorders: From the clinic to mice and back. J. Clin. Investig..

[13] Alexandrou A., Papaevripidou I., Alexandrou I.M., Theodosiou A., Evangelidou P., Kousoulidou L., Tanteles G., Christophidou-Anastasiadou V., Sismani C. (2019). De novo mosaic MECP2 mutation in a female with Rett syndrome. Clin. Case Rep..

[14] Wan M., Lee S.S.J., Zhang X., Houwink-Manville I., Song H.-R., Amir R.E., Budden S., Naidu S., Pereira J.L.P., Lo I.F. (1999). Rett Syndrome and beyond: Recurrent spontaneous and familial MECP2 mutations at CpG hotspots. Am. J. Hum. Genet..

[15] Gold W.A., Krishnarajy R., Ellaway C., Christodoulou J. (2018). Rett Syndrome: A Genetic Update and Clinical Review Focusing on Comorbidities. ACS Chem. Neurosci..

[16] Palmieri M., Pozzer D., Landsberger N. (2023). Advanced genetic therapies for the treatment of Rett syndrome: State of the art and future perspectives. Front. Neurosci..

[17] Downs J., Wong K., Leonard H. (2024). Associations between genotype, phenotype and behaviours measured by the Rett syndrome behaviour questionnaire in Rett syndrome. J. Neurodev. Disord..

[18] Kay C., Leonard H., Smith J., Wong K., Downs J. (2023). Genotype and sleep independently predict mental health in Rett syndrome: An observational study. J. Med. Genet..

[19] Boban S., Wong K., Epstein A., Anderson B., Murphy N., Downs J., Leonard H. (2016). Determinants of sleep disturbances in Rett syndrome: Novel findings in relation to genotype. Am. J. Med. Genet. Part A.

[20] Zito A., Lee J.T. (2024). Variable expression of MECP2, CDKL5, and FMR1 in the human brain: Implications for gene restorative therapies. Proc. Natl. Acad. Sci. USA.

[21] Hagberg B. (2002). Clinical manifestations and stages of rett syndrome. Ment. Retard. Dev. Disabil. Res. Rev..

[22] Naidu S., Murphy M., Moser H.W., Rett A., Opitz J.M., Reynolds J.F. (1986). Rett syndrome—natural history in 70 cases. Am. J. Med. Genet..

[23] Lee J., Leonard H., Piek J., Downs J. (2013). Early development and regression in Rett syndrome. Clin. Genet..

[24] Wong K., Leonard H., Jacoby P., Ellaway C., Downs J. (2015). The trajectories of sleep disturbances in Rett syndrome. J. Sleep Res..

[25] Halbach N., Smeets E., Steinbusch C., Maaskant M., van Waardenburg D., Curfs L. (2013). Aging in Rett syndrome: A longitudinal study. Clin. Genet..

[26] Tascini G., Dell’ISola G.B., Mencaroni E., Di Cara G., Striano P., Verrotti A. (2022). Sleep Disorders in Rett Syndrome and Rett-Related Disorders: A Narrative Review. Front. Neurol..

[27] Leven Y., Wiegand F., Wilken B. (2020). Sleep Quality in Children and Adults with Rett Syndrome. Neuropediatrics.

[28] Boban S., Leonard H., Wong K., Wilson A., Downs J. (2018). Sleep disturbances in Rett syndrome: Impact and management including use of sleep hygiene practices. Am. J. Med. Genet. Part A.

[29] Zhang X., Smits M., Curfs L., Spruyt K. (2024). Sleep and the Social Profiles of Individuals With Rett Syndrome. Pediatr. Neurol..

[30] Huang C.-H., Wong L.-C., Chu Y.-J., Hsu C.-J., Wang H.-P., Tsai W.-C., Lee W.-T. (2024). The sleep problems in individuals with Rett syndrome and their caregivers. Autism.

[31] Gold W.A., Percy A.K., Neul J.L., Cobb S.R., Pozzo-Miller L., Issar J.K., Ben-Zeev B., Vignoli A., Kaufmann W.E. (2024). Rett syndrome. Nat. Rev. Dis. Prim..

[32] Piazza C.C., Fisher W., Moser H. (1991). Behavioral treatment of sleep dysfunction in patients with the rett syndrome. Brain Dev..

[33] Dragich J.M., Kim Y., Arnold A.P., Schanen N.C. (2007). Differential distribution of the MeCP2 splice variants in the postnatal mouse brain. J. Comp. Neurol..

[34] Zhou Z., Hong E.J., Cohen S., Zhao W.-N., Ho H.-Y.H., Schmidt L., Chen W.G., Lin Y., Savner E., Griffith E.C. (2006). Brain-Specific Phosphorylation of MeCP2 Regulates Activity-Dependent Bdnf Transcription, Dendritic Growth, and Spine Maturation. Neuron.

[35] Ramirez J.-M., Karlen-Amarante M., Wang J.-D.J., Huff A., Burgraff N. (2022). Breathing disturbances in Rett syndrome. Handbook of Clinical Neurology.

[36] Lugaresi E., Cirignotta F., Montagna P. (1985). Abnormal breathing in the Rett syndrome. Brain Dev..

[37] Weese-Mayer D.E., Lieske S.P., Boothby C.M., Kenny A.S., Bennett H.L., Ramirez J. (2008). Autonomic dysregulation in young girls with Rett Syndrome during nighttime in-home recordings. Pediatr. Pulmonol..

[38] Amaddeo A., De Sanctis L., Arroyo J.O., Khirani S., Bahi-Buisson N., Fauroux B. (2019). Polysomnographic findings in Rett syndrome. Eur. J. Paediatr. Neurol..

[39] Bassett E., Heinle R., Johnston D. (2016). Sleep Apnea in Patients With Rett Syndrome: Roles for Polysomnography and Adenotonsillectomy. J. Child. Neurol..

[40] Patel A.A., Glaze D.G. (2020). Sleep and sleep disorders in Rett syndrome. Neurological Modulation of Sleep.

[41] Mackay J., Downs J., Wong K., Heyworth J., Epstein A., Leonard H. (2017). Autonomic breathing abnormalities in Rett syndrome: Caregiver perspectives in an international database study. J. Neurodev. Disord..

[42] Glaze D.G., Percy A.K., Skinner S., Motil K.J., Neul J.L., Barrish J.O., Lane J.B., Geerts S.P., Annese F., Graham J. (2010). Epilepsy and the natural history of Rett syndrome. Neurology.

[43] Dolce A., Ben-Zeev B., Naidu S., Kossoff E.H. (2013). Rett syndrome and epilepsy: An update for child neurologists. Pediatr. Neurol..

[44] Bricker K., Vaughn B.V. (2024). Rett syndrome: A review of clinical manifestations and therapeutic approaches. Front. Sleep.

[45] Nissenkorn A., Gak E., Vecsler M., Reznik H., Menascu S., Ben Zeev B. (2010). Epilepsy in Rett syndrome—The experience of a National Rett Center. Epilepsia.

[46] Mendoza J., Downs J., Wong K., Leonard H. (2021). Determinants of quality of life in Rett syndrome: New findings on associations with genotype. J. Med. Genet..

[47] Kaufmann W.E., Percy A.K., Neul J.L., Downs J., Leonard H., Nues P., Sharma G.D., Bartolotta T.E., Townend G.S., Curfs L.M.G. (2024). Burden of illness in Rett syndrome: Initial evaluation of a disorder-specific caregiver survey. Orphanet J. Rare Dis..

[48] Merbler A.M., Byiers B.J., Garcia J.J., Feyma T.J., Symons F.J. (2018). The feasibility of using actigraphy to characterize sleep in Rett syndrome. J. Neurodev. Disord..

[49] Byiers B.J., Merbler A.M., Burkitt C.C., Symons F.J. (2025). Challenges in Using Parent-Reported Bed and Wake Times for Actigraphy Scoring in Rett-Related Syndromes. Am. J. Intellect. Dev. Disabil..

[50] Sarber K.M., Howard J.J.M., Dye T.J., Pascoe J.E., Simakajornboon N. (2019). Sleep-Disordered Breathing in Pediatric Patients With Rett Syndrome. J. Clin. Sleep Med..

[51] Zhang X.-Y., Spruyt K. (2022). Literature Cases Summarized Based on Their Polysomnographic Findings in Rett Syndrome. Int. J. Environ. Res. Public Health.

[52] Carotenuto M., Esposito M., D’aNiello A., Rippa C.D., Precenzano F., Pascotto A., Bravaccio C., Elia M. (2013). Polysomnographic findings in Rett syndrome: A case–control study. Sleep Breath..

[53] Ammanuel S., Chan W.C., Adler D.A., Lakshamanan B.M., Gupta S.S., Ewen J.B., Johnston M.V., Marcus C.L., Naidu S., Kadam S.D. (2015). Heightened Delta Power during Slow-Wave-Sleep in Patients with Rett Syndrome Associated with Poor Sleep Efficiency. PLoS ONE.

[54] Davis P., Takach K., Maski K., Levin A. (2022). A circuit-level biomarker of Rett syndrome based on ectopic phase-amplitude coupling during slow-wave-sleep. Cereb. Cortex.

[55] Rodrigues G.D., Cordani R., Veneruso M., Chiarella L., Prato G., Ferri R., Carandina A., Tobaldini E., Nobili L., Montano N. (2024). Predominant cardiac sympathetic modulation during wake and sleep in patients with Rett syndrome. Sleep Med..

[56] Furqan M. (2023). Trofinetide—A new chapter in rett syndrome’s treatment. Front. Pharmacol..

[57] Lopes A.G., Loganathan S.K., Caliaperumal J. (2024). Rett Syndrome and the Role of MECP2: Signaling to Clinical Trials. Brain Sci..

[58] Percy A.K., Ananth A., Neul J.L. (2024). Rett Syndrome: The Emerging Landscape of Treatment Strategies. CNS Drugs.

[59] Camillo L., Pozzi M., Bernardo P., Pisano S., Nobile M. (2024). Profile of Trofinetide in the Treatment of Rett Syndrome: Design, Development and Potential Place in Therapy. Drug Des. Dev. Ther..

[60] Bricker K., Vaughn B.V. (2024). Review of Sleep Disorders and Therapeutic Approaches in Patients With Autism Spectrum Disorder and Rett Syndrome. Sleep Med. Res..

[61] Fu C., Armstrong D., Marsh E., Lieberman D., Motil K., Witt R., Standridge S., Nues P., Lane J., Dinkel T. (2020). Consensus guidelines on managing Rett syndrome across the lifespan. BMJ Paediatr. Open.

[62] International Rett Syndrome Foundation (2024). Rett Syndrome: Comprehensive Care Guidelines. https://www.rettsyndrome.org/wp-content/uploads/2024/12/Comprehensive-Care-Guidelines.pdf.

[63] Blackmer A.B., Feinstein J.A. (2016). Management of Sleep Disorders in Children With Neurodevelopmental Disorders: A Review. Pharmacother. J. Hum. Pharmacol. Drug Ther..

[64] Buckley A.W., Hirtz D., Oskoui M., Armstrong M.J., Batra A., Bridgemohan C., Coury D., Dawson G., Donley D., Findling R.L. (2020). Practice guideline: Treatment for insomnia and disrupted sleep behavior in children and adolescents with autism spectrum disorder: Report of the Guideline Development, Dissemination, and Implementation Subcommittee of the American Academy of Neurology. Neurology.

[65] Grigg-Damberger M., Ralls F. (2013). Treatment strategies for complex behavioral insomnia in children with neurodevelopmental disorders. Curr. Opin. Pulm. Med..

[66] Jan J.E., Owens J.A., Weiss M.D., Johnson K.P., Wasdell M.B., Freeman R.D., Ipsiroglu O.S. (2008). Sleep hygiene for children with neurodevelopmental disabilities. Pediatrics.

[67] Epstein A., Leonard H., Davis E., Williams K., Reddihough D., Murphy N., Whitehouse A., Downs J. (2016). Conceptualizing a quality of life framework for girls with Rett syndrome using qualitative methods. Am. J. Med. Genet. Part A.

[68] Woodford E.C., France K.G., Blampied N.M., Hanning U., Swan C.E., McLay L.K. (2024). Behavioral Sleep Interventions for Children with Rare Genetic Neurodevelopmental Conditions: A Retrospective Analysis of Overall Outcomes for 26 Cases. Adv. Neurodev. Disord..

[69] McArthur A.J., Budden S.S. (1998). Sleep dysfunction in Rett syndrome: A trial of exogenous melatonin treatment. Dev. Med. Child. Neurol..

[70] Cohen P.A., Avula B., Wang Y.-H., Katragunta K., Khan I. (2023). Quantity of Melatonin and CBD in Melatonin Gummies Sold in the US. JAMA.

[71] Owens J.A., Rosen C.L., Mindell J.A., Kirchner H.L. (2010). Use of pharmacotherapy for insomnia in child psychiatry practice: A national survey. Sleep Med..

[72] Bruni O., Angriman M., Calisti F., Comandini A., Esposito G., Cortese S., Ferri R. (2018). Practitioner Review: Treatment of chronic insomnia in children and adolescents with neurodevelopmental disabilities. J. Child. Psychol. Psychiatry.

[73] Ellaway C.J., Peat J., Williams K., Leonard H., Christodoulou J. (2001). Medium-term open label trial of L-carnitine in Rett syndrome. Brain Dev..

[74] Motil K.J., Caeg E., Barrish J.O., Geerts S., Lane J.B., Percy A.K., Annese F., McNair L., Skinner S.A., Lee H. (2012). Gastrointestinal and nutritional problems occur frequently throughout life in girls and women with rett syndrome. J. Pediatr. Gastroenterol. Nutr..

[75] Brook E., Usman M. (2015). SSRIs in Rett syndrome. Aust. New Zealand J. Psychiatry.

[76] Picchietti D.L., Bruni O., de Weerd A., Durmer J.S., Kotagal S., Owens J.A., Simakajornboon N. (2013). Pediatric restless legs syndrome diagnostic criteria: An update by the International Restless Legs Syndrome Study Group. Sleep Med..

[77] Killian W., Riederer P., Linkesch W. (1987). Serum iron status in Rett syndrome. Brain Dev..

[78] Winkelman J.W., Berkowski J.A., DelRosso L.M., Koo B.B., Scharf M.T., Sharon D., Zak R.S., Kazmi U., Falck-Ytter Y., Shelgikar A.V. (2025). Treatment of restless legs syndrome and periodic limb movement disorder: An American Academy of Sleep Medicine clinical practice guideline. J. Clin. Sleep Med..

[79] Cherchi C., Chiappini E., Amaddeo A., Testa M.B.C., Banfi P., Veneselli E., Cutrera R. (2024). Management of respiratory issues in patients with Rett syndrome: Italian experts’ consensus using a Delphi approach. Pediatr. Pulmonol..

[80] Toward M.A., Abdala A.P., Knopp S.J., Paton J.F.R., Bissonnette J.M. (2013). Increasing brain serotonin corrects CO2 chemosensitivity in methyl-CpG-binding protein 2 (Mecp2)-deficient mice. Exp. Physiol..

[81] Gökben S., Ardıç Ü.A., Serdaroğlu G. (2012). Use of Buspirone and Fluoxetine for Breathing Problems in Rett Syndrome. Pediatr. Neurol..

[82] Ohno K., Saito Y., Ueda R., Togawa M., Ohmae T., Matsuda E., Fujiyama M., Maegaki Y. (2016). Effect of Serotonin 1A Agonists and Selective Serotonin Reuptake Inhibitors on Behavioral and Nighttime Respiratory Symptoms in Rett Syndrome. Pediatr. Neurol..

[83] Abdala A.P.L., Dutschmann M., Bissonnette J.M., Paton J.F.R. (2010). Correction of respiratory disorders in a mouse model of Rett syndrome. Proc. Natl. Acad. Sci. USA.

[84] Tarquinio D.C., Hou W., Neul J.L., Kaufmann W.E., Glaze D.G., Motil K.J., Skinner S.A., Lee H.-S., Percy A.K. (2015). The Changing Face of Survival in Rett Syndrome and MECP2-Related Disorders. Pediatr. Neurol..

[85] Spruyt K., Curfs L.M.G. (2014). Non-pharmacological management of problematic sleeping in children with developmental disabilities. Dev. Med. Child. Neurol..

[86] Hung P.L., Wu K.L.H., Chen C.J., Siu K.K., Hsin Y.J., Wang L.J., Wang F.S. (2021). Music-Based Intervention Ameliorates Mecp2-Loss-Mediated Sociability Repression in Mice through the Prefrontal Cortex FNDC5/BDNF Pathway. Int. J. Mol. Sci..

